# Supplementary material for: Supernumerary B chromosomes of Aegilops speltoides undergo precise elimination in roots early in embryo development
Source: Nat Commun. 2020 Jun 2;11:2764. doi: 10.1038/s41467-020-16594-x (PMC7265534; doi:10.1038/s41467-020-16594-x)
Supplement: Supplementary file 1 — Supplementary Information [file 41467_2020_16594_MOESM1_ESM.pdf]

Supernumerary B chromosomes of *Aegilops speltoides* undergo precise elimination in roots early in embryo development

Ruban *et al.*

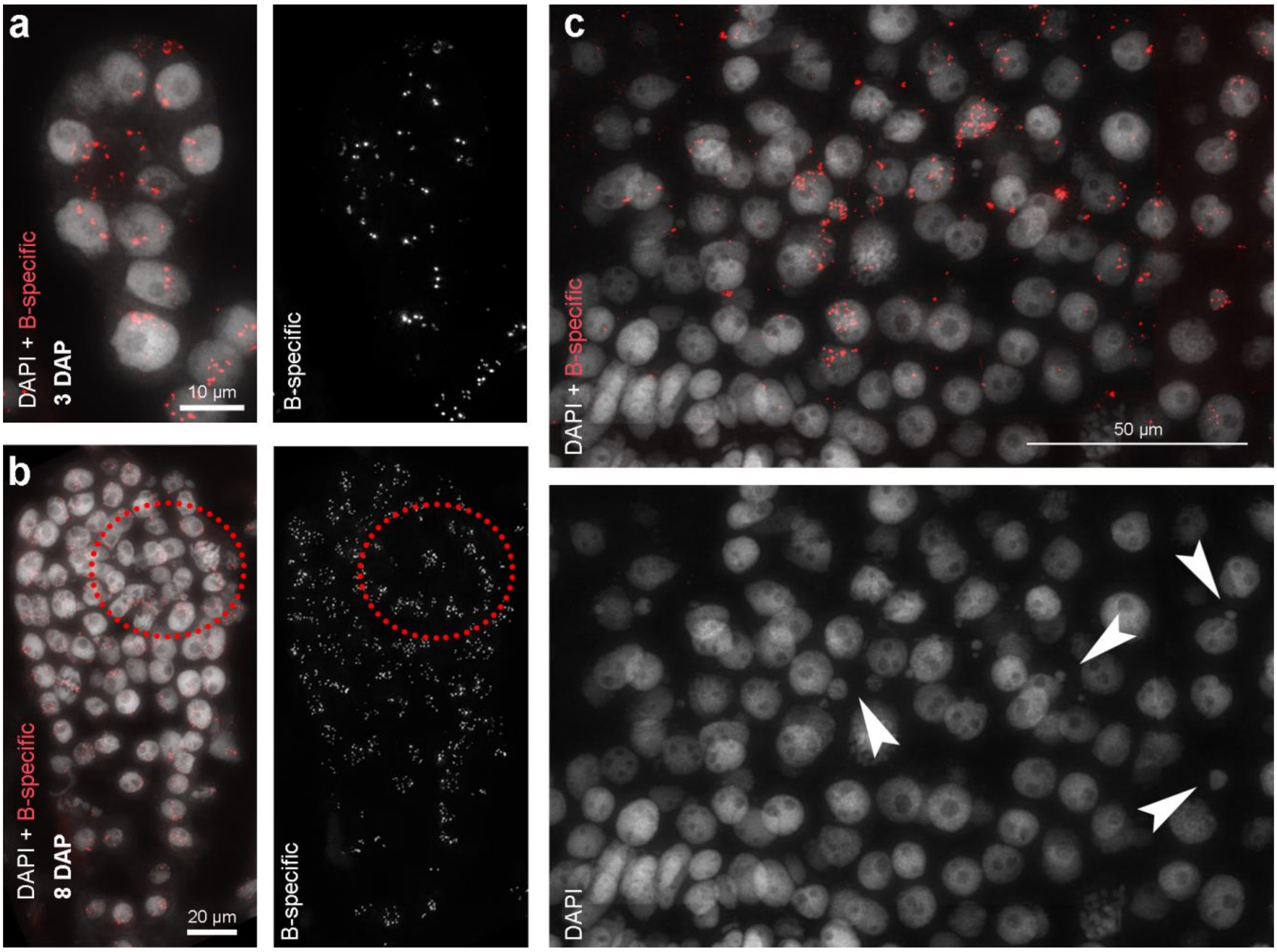

Supplementary Figure 1. Elimination of B chromosomes starts at the onset of embryo differentiation of *Ae. speltoides*. (a) No elimination of Bs in 3 DAP-old embryos was observed, as the signals of the B-specific FISH probe AesTR-183 (in red) were present in all nuclei. (b) 8 DAP old embryo shows beginning of B chromosome elimination. The region where elimination already started is marked by the red dotted circle. (c) Formation of B-positive micronuclei (arrowed). Nuclei are stained with DAPI (in grey). For each stage of embryo development 2 to 4 embryos were sectioned and analysed. Obtained results were consistent.

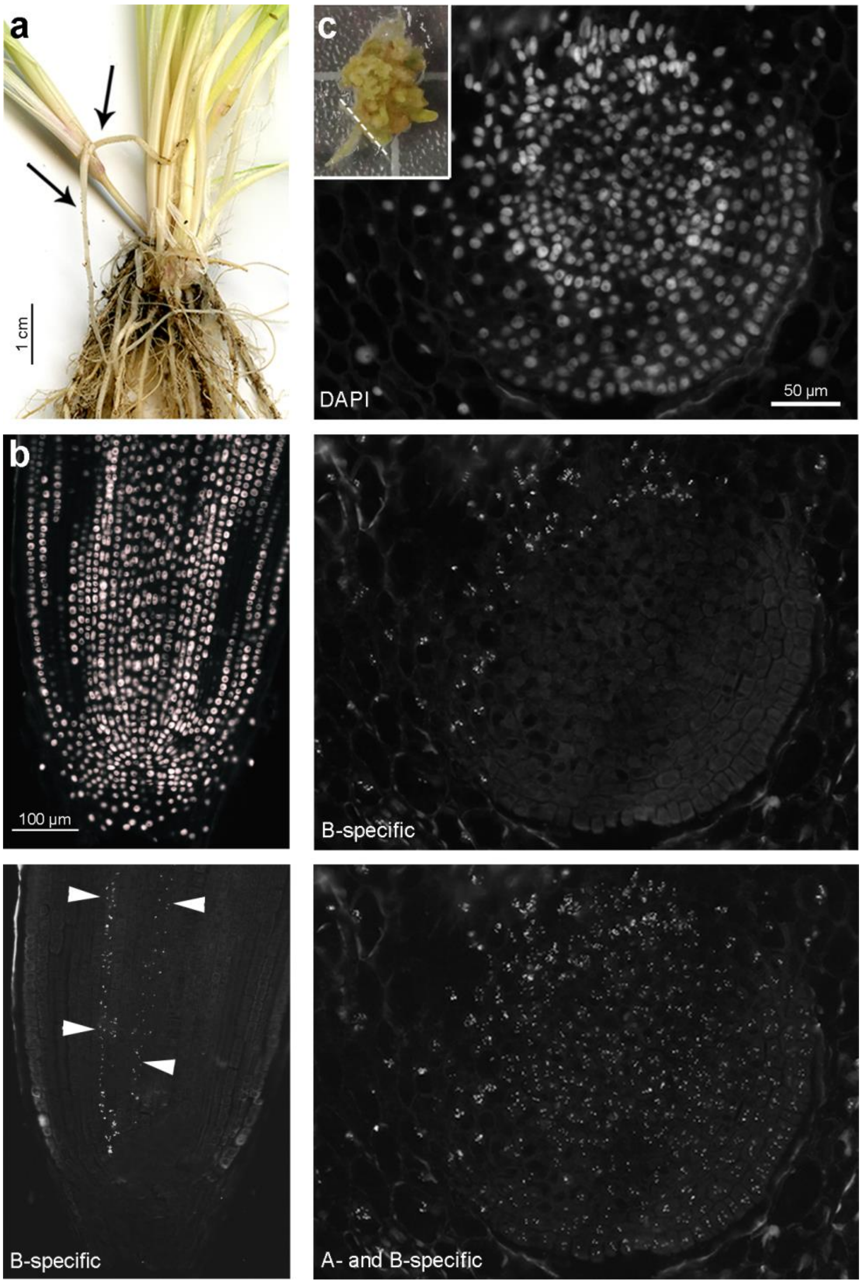

Supplementary Figure 2. Elimination of B chromosomes in different root types. (a) Tillering node of an *Ae. speltoides* plant. Arrows indicate adventitious roots on one of the tillers. (b) Longitudinal tissue section of an adventitious root. The Bs are labeled by the B-specific FISH probe AesTR-183, nuclei are stained by DAPI. B-carrying cell lineages are indicated by arrowheads. Sections were made from 4 roots and observed results were consistent. (c) Transversal tissue section of a root from an organogenic callus. The insert shows the callus with the sectioning plane marked by a dashed line. The Bs are labeled by the B-specific probe AesTR-183. The probe AesTR-148 which localizes on As and Bs was used as a control. Nuclei are stained by DAPI. Note the absence of B-specific signals in the root area. Sections were made from calli derived from 10 embryos. Observed results were consistent.

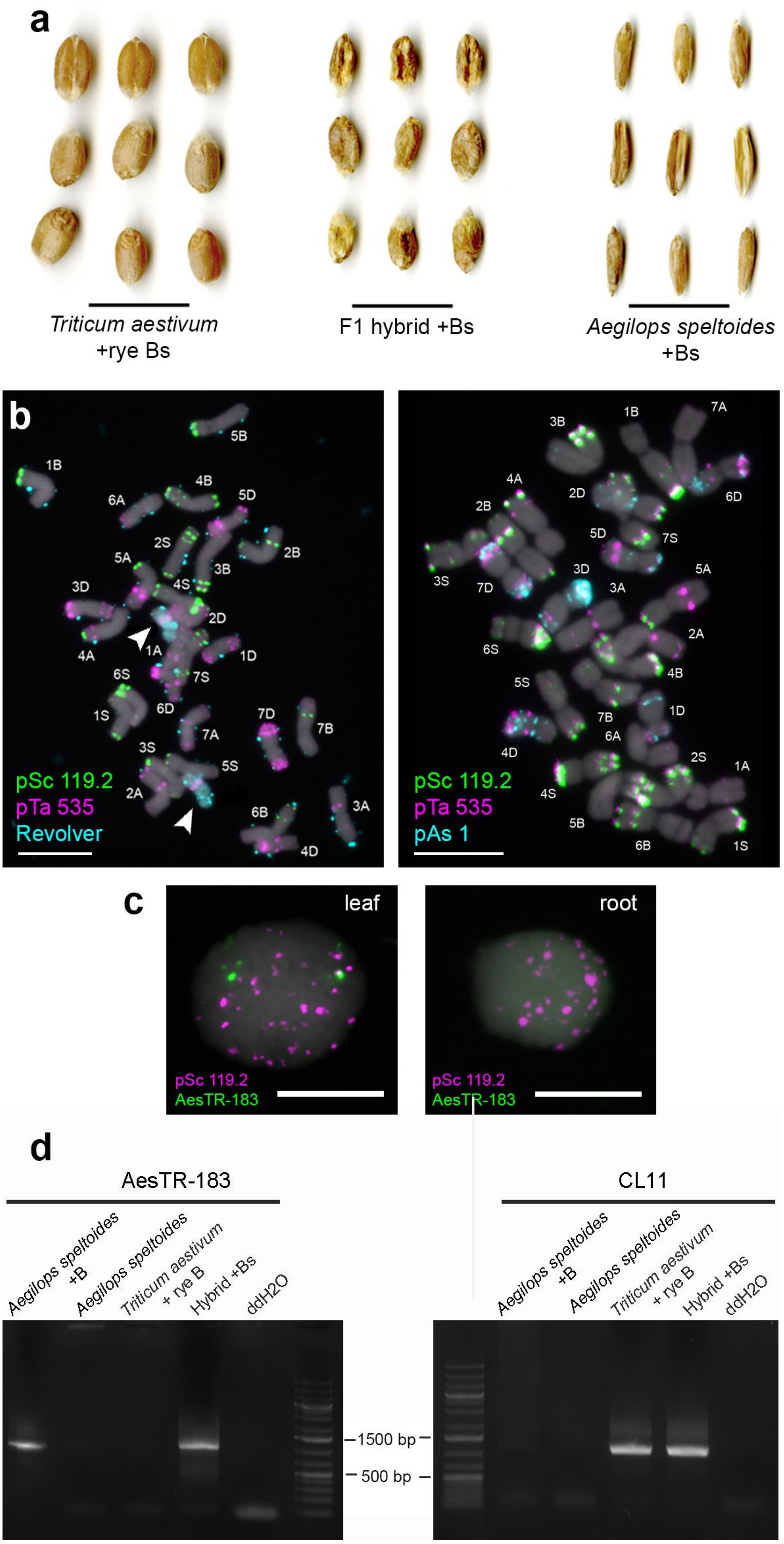
a
Triticum aestivum
+rye Bs
F1 hybrid +Bs
Aegilops speltoides
+Bs
b
pSc 119.2
pTa 535
Revolver
pSc 119.2
pTa 535
pAs 1
c
leaf
root
pSc 119.2
AesTR-183
pSc 119.2
AesTR-183
d
AesTR-183
CL11
Aegilops speltoides +B
Aegilops speltoides
Triticum aestivum + rye B
Hybrid +Bs
ddH2O
1500 bp
500 bp

Supplementary Figure 3. The mechanism responsible for the elimination of *Ae. speltoides* Bs works in a hybrid background as well but does not affect the coexisting Bs of rye. *Ae. speltoides* B chromosomes were not found in the roots of the F1 hybrids between *T. aestivum* containing additional rye Bs and *Ae. speltoides* also containing Bs. However, rye Bs were present in leaves as well as in roots of the F1 plants. This was observed for 17 F1 hybrids which were analysed by FISH and PCR. One plant had no Bs, 16 contained Bs of *Ae. speltoides* only and 4 possessed Bs from *Ae. speltoides* and rye. (a) Grains of *T. aestivum* + rye Bs, the F1 hybrid, and *Ae. speltoides* +Bs. Scale bar, 1 cm. (b) Root metaphase chromosomes of F1 hybrids with *Ae. speltoides* and rye Bs (left) and F1 hybrids with only *Ae. speltoides* Bs (right). In both cases *Ae. speltoides* Bs were absent in the root cells. Rye Bs (arrowheads) were labelled with the rye-specific mobile element Revolver (in cyan). The probes pSc 119.2 (in green), pTa 535 (in purple) and pAs 1 (in cyan) were used for chromosome identification. Chromatin was stained by DAPI (in grey). Scale bars, 10 µm. (c) Leaf and root nuclei of an F1 hybrid containing *Ae. speltoides* Bs. The presence of Bs is indicated by the signals of the B-specific FISH probe AesTR-183 (in green) and the probe pSc 119.2 (in purple) was used as a control. Chromatin was stained by DAPI (in grey). Scale bars, 10 µm. Nuclei from 3 F1 hybrids (100 nuclei each) were analysed. (d) PCR amplification of the *Ae. speltoides* B-specific repeat AesTR-183 and the rye B-specific repeat CL11 using DNA isolated from leaf tissue of *Ae. speltoides* +B, *Ae. speltoides* 0B, *T. aestivum* + rye B, and F1 hybrid. Water was used instead of DNA as a negative control. Marker GeneRuler 1 kb Plus DNA ladder. This PCR analysis was routinely used to determine presence of Bs. Results were confirmed by FISH analysis. Source data underlying Supplementary Figure 3d are provided as a Source Data file.

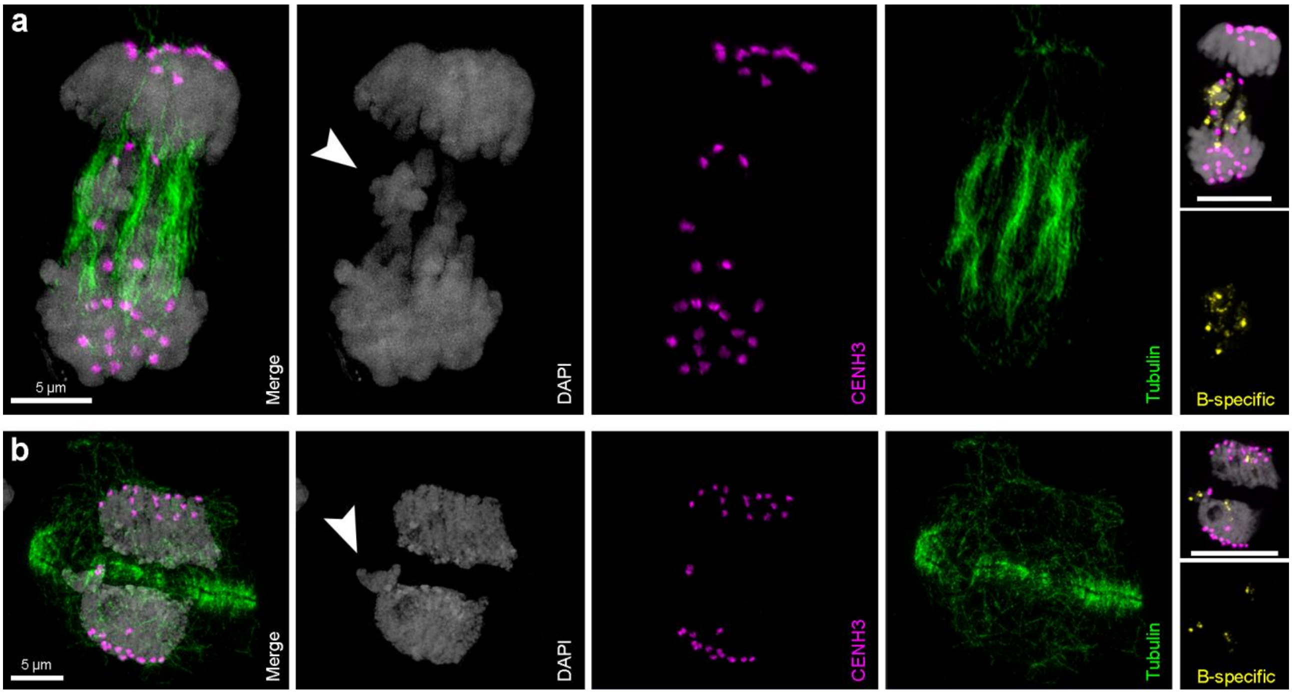

Supplementary Figure 4. Nondisjunction of centromere-active B chromosomes results in the formation of micronuclei. Lagging of Bs and retention of B-chromatin close to the cell equator occurred. Immunostaining of CENH3 (in purple) and α-tubulin (in green). FISH signals of the B-specific probes AesTR-183 and AesTR-205 are shown in yellow. Chromatin was stained with DAPI (in blue). (a) Lagging B chromatin in between As at late anaphase. In total 7 anaphase cells with lagging Bs were analysed in 3 embryos using super resolution microscopy. (b) The phragmoplast (green) formation is impaired at telophase due to the lagging the B chromosomes. It was observed in 3 embryos in 5 telophases.

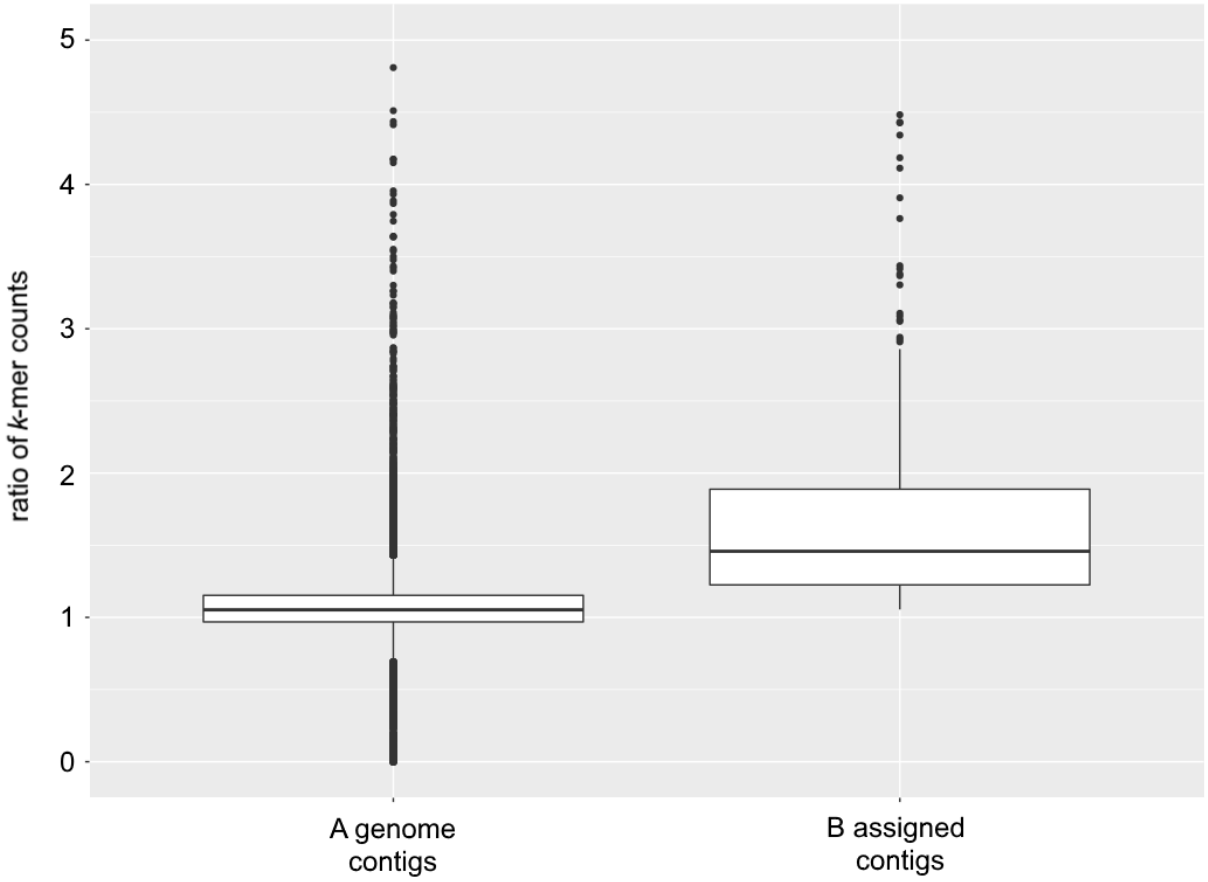

Supplementary Figure 5. *K*-mer ratio plot of A genomic and B chromosome assigned contigs. The average *k*-mer counts per gene was calculated for the 0B and the +B dataset and the ratio (+B/0B) was plotted for both subsets. A total of N=28.516 contigs were analysed from the A genome and a total of N=187 contigs were analysed from B chromosome. Boxplot details are given by number summary for A genome contigs (A) and B assigned contigs (B). Upper whisker values are 1.43 (A) and 3.11 (B). Maximum (3rd quartile) values are 1.15 (A) and 2.01 (B). Median values are 1.05 (A) and 1.48 (B). Minimum (1st quartile) values are 0.97 (A) and 1.23 (B). Lower whisker values are 0.69 (A) and 1.06 (B). Minimum values are 0.00 (A) and 1.06 (B). Maximum values are 13.61 (A) and 17.50 (B). Source data are provided as a Source Data file.

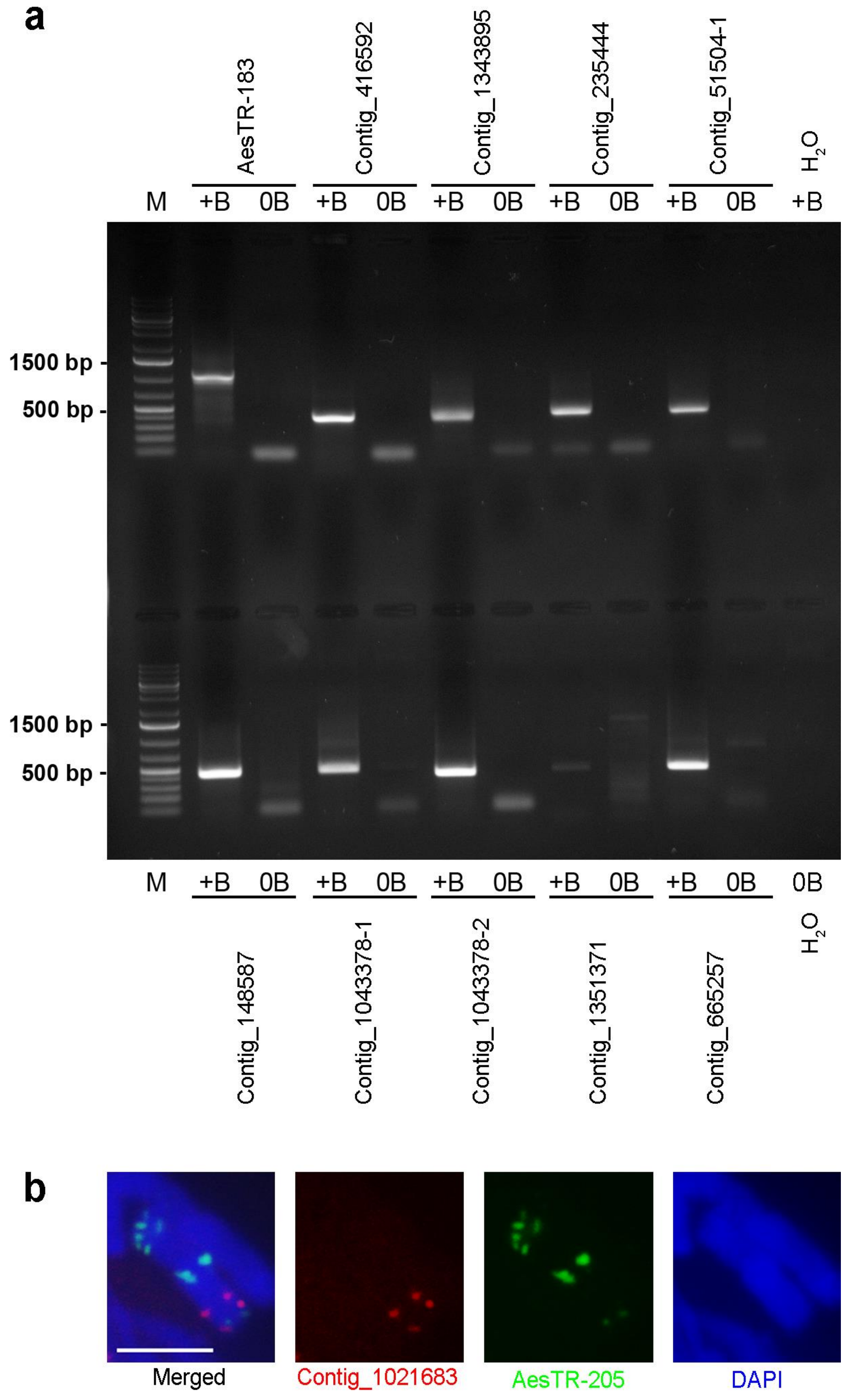

Supplementary Figure 6. Validation of B-specificity of selected sequences. (a) Confirmation of the *in silico* identified B-specific sequences using +B and 0B genomic DNA of *Ae. speltoides* as template. Primer pairs are provided in Supplementary Table 9. Previously identified B-specific repeat AesTR-183 was used as a control. PCR was repeated for 3 times with consistent results. Source data are provided as a Source Data file. (b) FISH with the probe designed for predicted B-specific short repeat identified in Contig_1021683 (in red) of *Ae. speltoides* assembly. Previously identified B-specific repeat AesTR-205 (in green) was used as a control. A selected B chromosome is shown. No signals were detected on A chromosomes. For confirmation, contig_1021683 probe was hybridized to chromosomes of 3 plants. Scale bar, 5 µm. Source data underlying Supplementary Figure 6a are provided as a Source Data file.

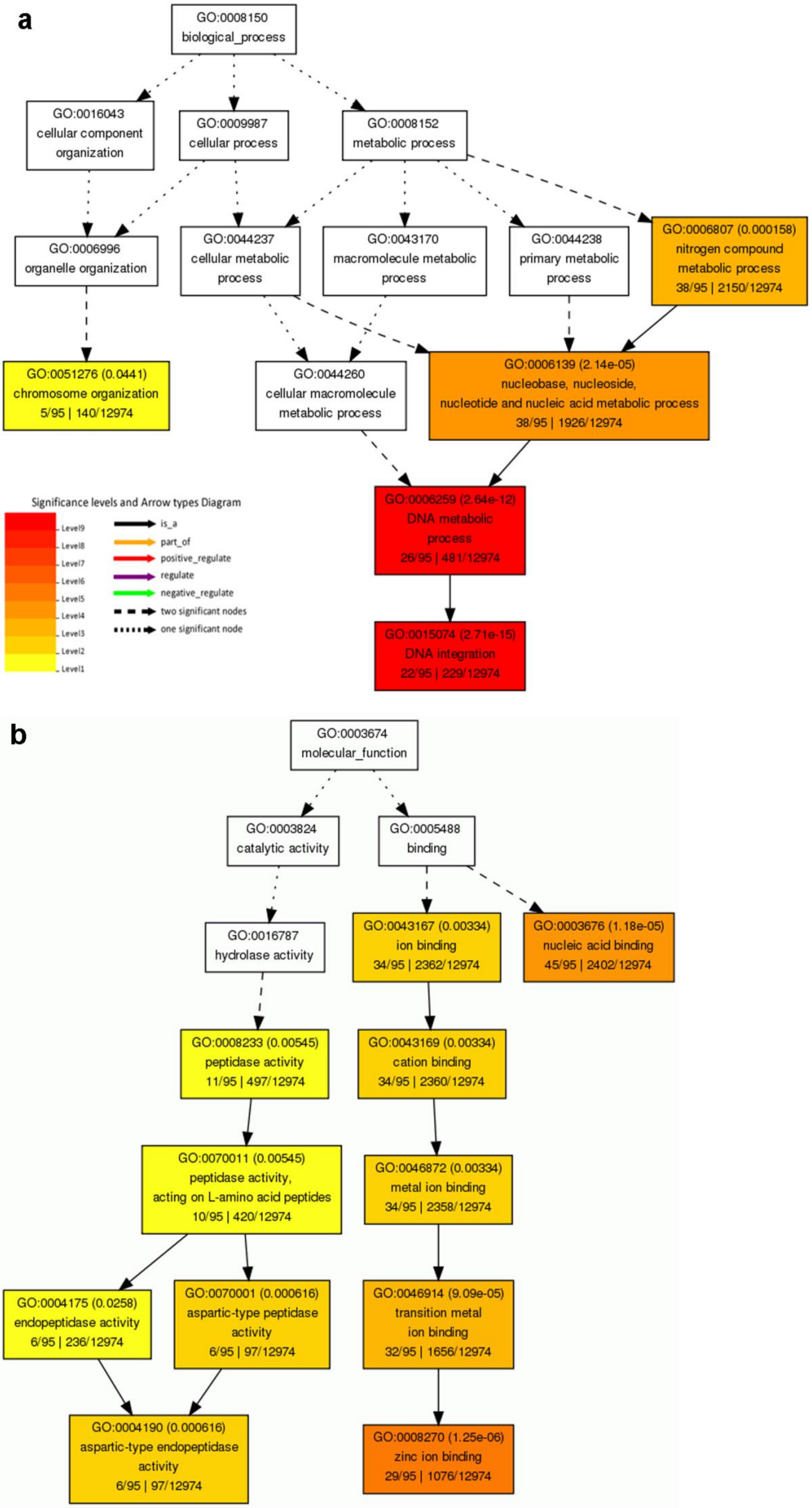
a
GO:0008150
biological_process
GO:0016043
cellular component organization
GO:0009987
cellular process
GO:0008152
metabolic process
GO:0006996
organelle organization
GO:0044237
cellular metabolic process
GO:0043170
macromolecule metabolic process
GO:0044238
primary metabolic process
GO:0006807 (0.000158)
nitrogen compound metabolic process
38/95 | 2150/12974
GO:0051276 (0.0441)
chromosome organization
5/95 | 140/12974
GO:0044260
cellular macromolecule metabolic process
GO:0006139 (2.14e-05)
nucleobase, nucleoside, nucleotide and nucleic acid metabolic process
38/95 | 1926/12974
Significance levels and Arrow types Diagram
Level9
Level8
Level7
Level6
Level5
Level4
Level3
Level2
Level1
is_a
part_of
positive_regulate
regulate
negative_regulate
two significant nodes
one significant node
GO:0006259 (2.64e-12)
DNA metabolic process
26/95 | 481/12974
GO:0015074 (2.71e-15)
DNA integration
22/95 | 229/12974
b
GO:0003674
molecular_function
GO:0003824
catalytic activity
GO:0005488
binding
GO:0016787
hydrolase activity
GO:0043167 (0.00334)
ion binding
34/95 | 2362/12974
GO:0003676 (1.18e-05)
nucleic acid binding
45/95 | 2402/12974
GO:0008233 (0.00545)
peptidase activity
11/95 | 497/12974
GO:0043169 (0.00334)
cation binding
34/95 | 2360/12974
GO:0070011 (0.00545)
peptidase activity, acting on L-amino acid peptides
10/95 | 420/12974
GO:0046872 (0.00334)
metal ion binding
34/95 | 2358/12974
GO:0004175 (0.0258)
endopeptidase activity
6/95 | 236/12974
GO:0070001 (0.000616)
aspartic-type peptidase activity
6/95 | 97/12974
GO:0046914 (9.09e-05)
transition metal ion binding
32/95 | 1656/12974
GO:0004190 (0.000616)
aspartic-type endopeptidase activity
6/95 | 97/12974
GO:0008270 (1.25e-06)
zinc ion binding
29/95 | 1076/12974

Supplementary Figure 7. The results of the GO term enrichment analysis based on the 229 candidates assigned to the B chromosome of *Ae. speltoides*. SEA tool of the agriGO v2.0 displays hierarchical tree graphs of statistically significant GO terms (adjusted p-value < 0.05) in (a) the biological process category and (b) in the molecular function category. The significant terms are colored, while non-significant terms are shown as white boxes. The degree of color positively correlates with the enrichment level of the GO term. The type of lines (solid, dashed, and dotted) reflects two, one and zero enriched terms at both ends connected by the line, respectively. The information within each box of the GO term includes GO term ID, adjusted p-value, description of the term, item number corresponding to the GO term in the query list and background, and the total number of query list and background.

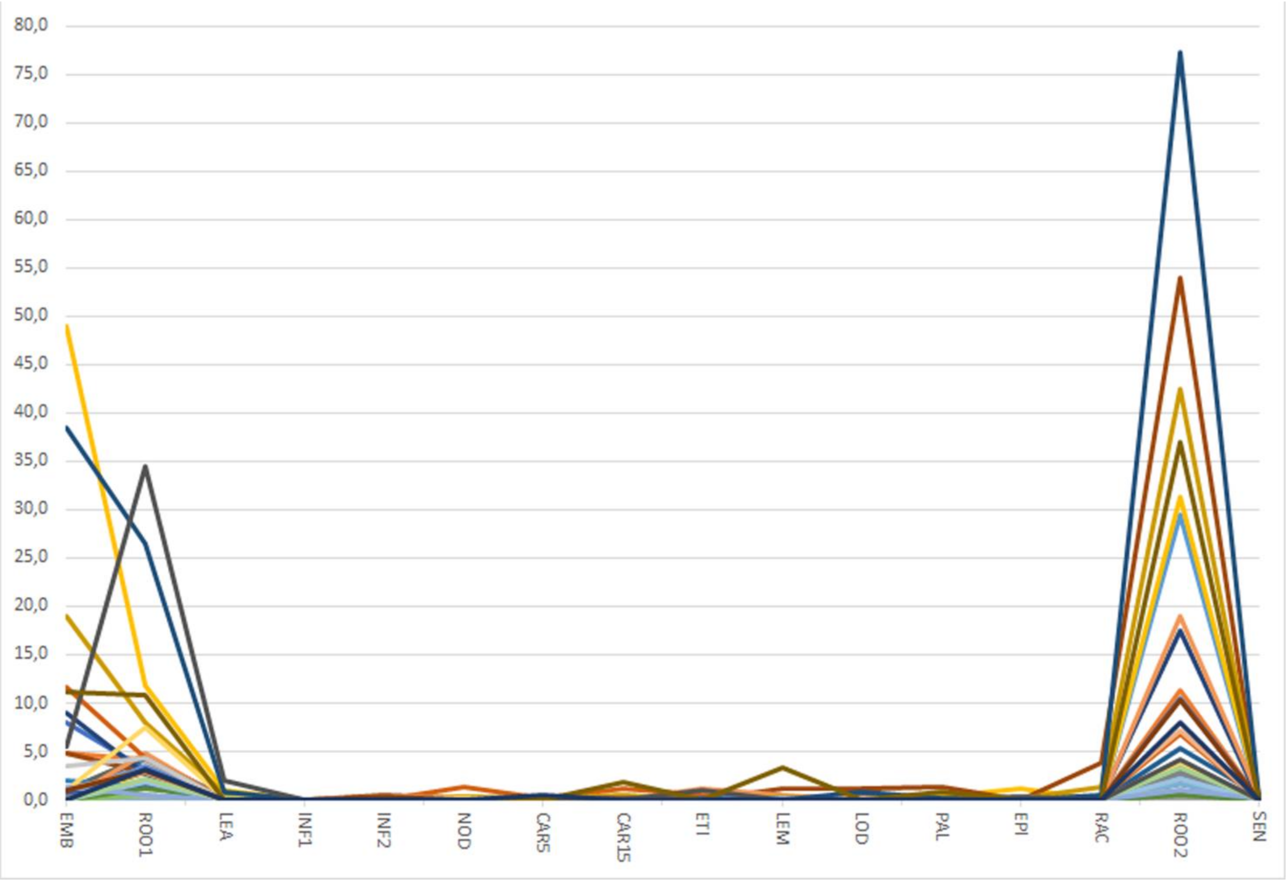

Supplementary Figure 8. Expression profile of barley genes across developmental stages based on the BARLEX database. B-chromosome related genes of *Ae.speltoides* (229) were aligned against all transcript sequences of high-confidence genes of barley (IPK Barley BLAST Server). The graph represents genes specifically expressed in roots. Details are shown in Supplementary Data 2 and 3. Values are in FPKM (Fragments per kilobase of exon model per million mapped reads). EMB, 4-day embryos; ROO1, roots from seedlings (10 cm shoot stage); LEA, shoots from seedlings (10 cm shoot stage); INF1, young developing inflorescences (5mm); INF2, developing inflorescences (1-1.5 cm); NOD, developing tillers, 3rd internode (42 DAP); CAR5, developing grain (5 DAP); CAR15, developing grain (15 DAP); ETI, etiolated seedling, dark cond. (10 DAP); LEM, inflorescences, lemma (42 DAP); LOD, inflorescences, lodicule (42 DAP); PAL, dissected inflorescences, palea (42 DAP); EPI, epidermal strips (28 DAP); RAC, inflorescences, rachis (35 DAP); ROO2, roots (28 DAP); SEN, senescing leaves (56 DAP).

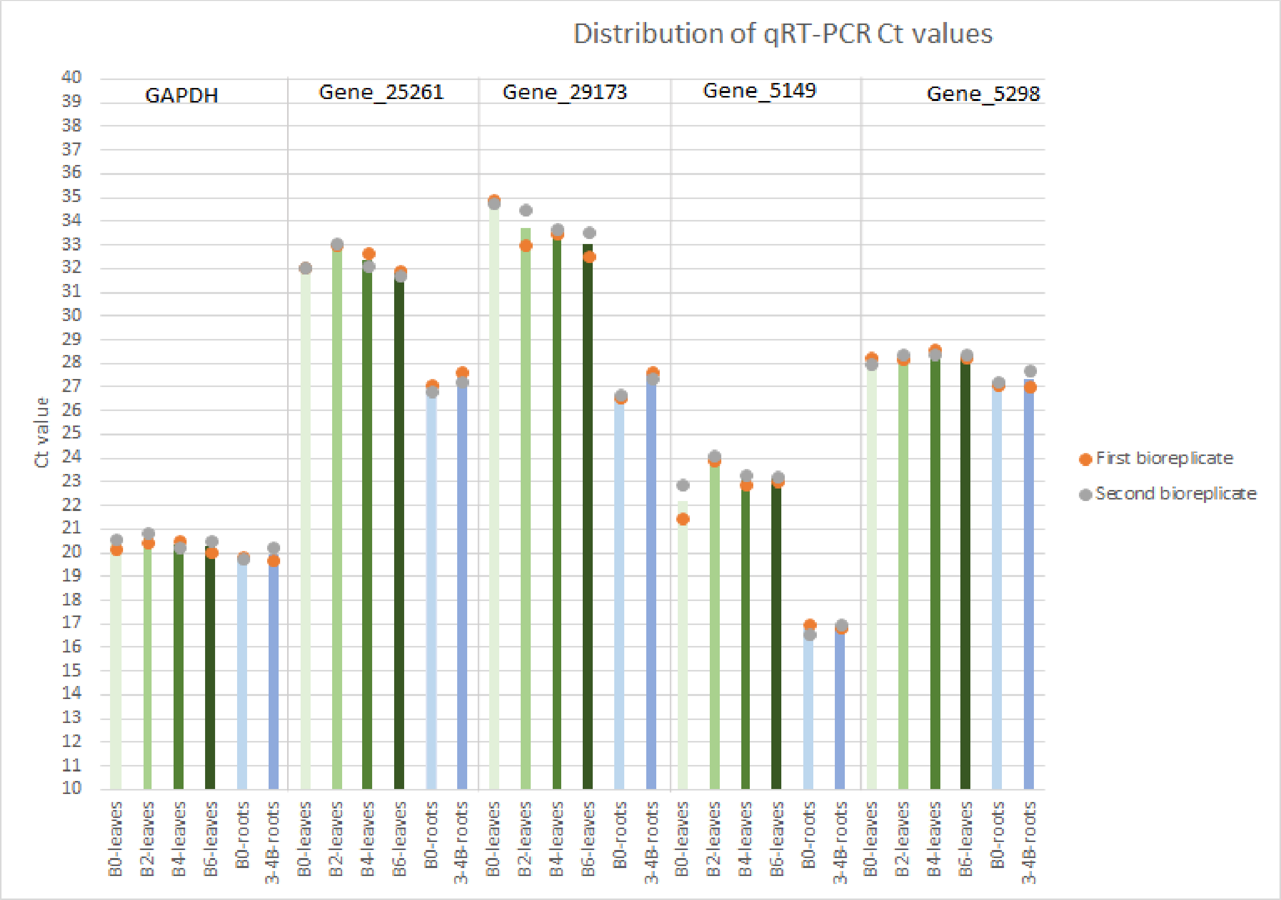

Supplementary Figure 9. Distribution of Ct values for four root-related genes across *Ae. speltoides* samples using qRT-PCR. Leaves and roots of plants with and without Bs were tested. GAPDH is used as a reference gene. Bars denote the mean value of the Ct per condition. Dots represent the mean Ct values per biological replicate. Source data are provided as a Source Data file.

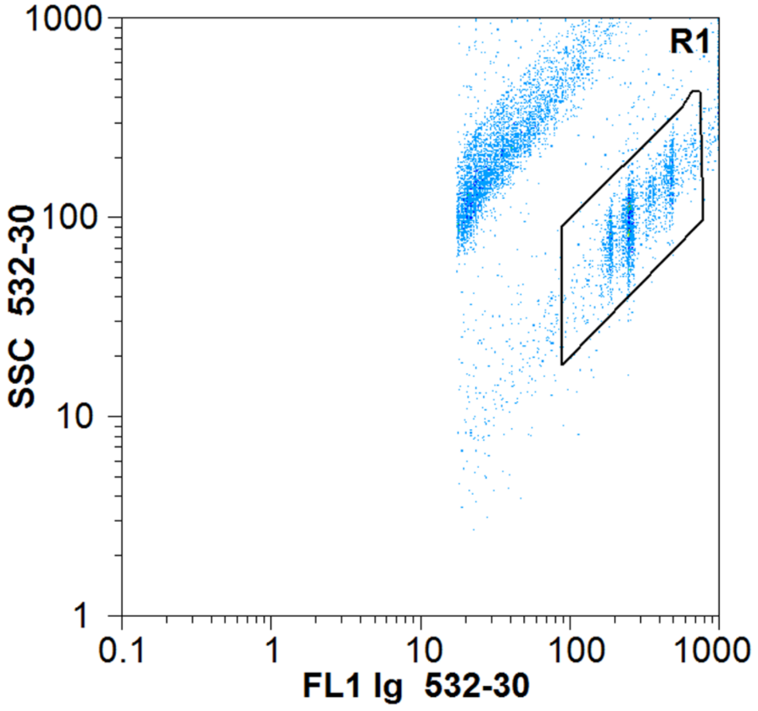

Supplementary Figure 10. Gating strategy for flow cytometric analysis. For all flow cytometric analysis the channel of the used DNA dye was set as trigger. In the primary plot of the fluorescence of the DNA dye versus side scatter the population of nuclei was gated (R1) and separated from cell debris.

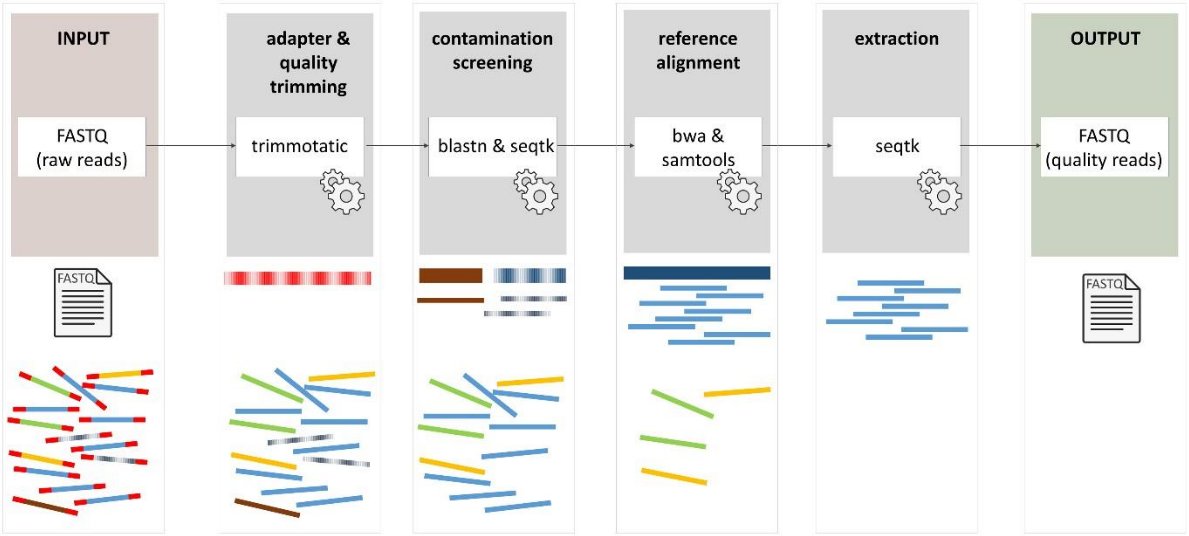

Supplementary Figure 11. Workflow of the MiDiSeq pipeline. Quality improvement workflow for microdissected Illumina sequenced reads. The MiDiSeq Pipeline is divided into 4 internal processing steps. First, adapter and low-quality sequences are removed from input data (e.g. Illumina sequencing adapter). Second, the data is screened for putative sequencing contamination as defined by the user (e.g. human or bacterial DNA or sequencing control spike-ins like PhiX). Third, pre-filtered reads are aligned to a user-defined reference sequence (e.g species of interest or species with phylogenetic close distance). Fourth, reads with confident alignments are extracted. All four steps can manually be defined by the user.

Supplementary Table 1. Details of raw sequence data.

dataset	sequencing method	type	reads [R1]	bp [R1]	reads [R2]	bp [R2]	Gbp	EBI/ENA
MiDiSeq	microdiss. Bs	B	12 027 933	1 202 793 300	12 027 933	1 202 793 300	2,41	**PRJEB29864**
total			12 027 933	1 202 793 300	12 027 933	1 202 793 300	2,41	
1111160	WGS	0B	63 486 231	6 412 109 331	63 486 231	6 412 109 331	12,82	**PRJEB29864**
1111162	WGS	0B	64 478 391	6 512 317 491	64 478 391	6 512 317 491	13,02	**PRJEB29864**
193936	WGS	0B	80 854 476	8 085 447 600	80 854 476	8 085 447 600	16,17	PRJEB29862
total			208 819 098	21 009 874 422	208 819 098	21 009 874 422	42,02	
1111161	WGS	+B	68 878 259	6 956 704 159	68 878 259	6 956 704 159	13,91	**PRJEB29864**
1111163	WGS	+B	69 912 496	7 061 162 096	69 912 496	7 061 162 096	14,12	**PRJEB29864**
193936	WGS	+B	90 508 134	9 050 813 400	90 508 134	9 050 813 400	18,10	PRJEB29862
total			229 298 889	23 068 679 655	229 298 889	23 068 679 655	46,14	
total sequence data			438 117 987		438 117 987		**88,16**	
genome coverage*							**14,77**	

The table lists sequencing characteristics of raw reads for microdissected and whole genome shotgun sequencing (0B and +B) including corresponding EBI/ENA project numbers. (*) The genome coverage calculation is based on a total genome size of 5.97 Gb (including B chromosome of 570 Mb) using WGS paired-end data.

Supplementary Table 2. B chromosome-assignment classes.

confidence class	class description	number of contigs	contigs [%]	range of values for sum of criteria weights
4	high	948	0,06	> 0.7
3	moderate	7 713	0,49	> 0.6 <=0.7
2	conservative	22 369	1,43	> 0.3 <=0.6
1	low	143 460	9,15	> 0.1 <=0.3
0	no assignment	1 393 218	88,87	>=0.0 <=0.1
total		1 567 708	100,00	0.0 - 1.0

The list describes the four assignment classes 1) low, 2) conservative, 3) moderate and 4) high confidence and corresponding results. The assignment relies on four defined criteria (‘MiDiSeq’, ‘FC5’, ‘AFC2’ and ‘KI33’) which are accumulated as a weighted sum. Details of confidence classes are given in Supplementary Table 3.

Supplementary Table 3. Details of assignment criteria and weights.

Threshold	Assignment confidence	#Contigs	Criteria [weights]			
			MiDiSeq5 [0.40]	FC5 [0.35]	AFC2 [0.15]	KI33 [0.10]
0,7	high	651	X	X	X	X
0,7	=	0	X	X	X	
0,7	=	106	X	X		X
0,7	=	191	X	X		
0,6	moderate	5 094	X		X	X
0,6	=	2 619		X	X	X
0,3	conservative	3 076	X			X
0,3	=	0	X		X	
0,3	=	18 025	X			
0,3	=	461		X		X
0,3	=	0		X	X	
0,3	=	807		X		
0,1	low	143 460			X	X
0,1	=	0			X	
0	no assignment	91 972				X
0	no assignment	1 301 246				

The table illustrates the different scenarios of observed criteria and corresponding assignment classes, including final number of contigs that are characterized by the respective features. The ‘x’ in the four criteria columns on the right denote that the criterium is fulfilled.

Supplementary Table 4. Assignment of the B chromosome-derived contigs to the B genome of hexaploid wheat.

chromosome	number of contigs	length of contigs [bp]	length of contigs [Mbp]	number of contigs with genes	number of genes in contigs
1	5 506	4 398 224	4,40	132	149
2	8 260	5 753 551	5,75	141	158
3	8 649	6 595 078	6,60	174	203
4	4 276	3 784 398	3,78	115	134
5	12 536	8 148 567	8,15	170	192
6	9 447	6 377 934	6,38	157	183
7	8 396	5 799 016	5,80	133	151
chrUn	4 188	2940271	2,94	61	66
NA	113 170	45695051	45,70	161	166
clDNA	1	35 264	0,04	1	14
mtDNA	61	219 471	0,22	8	20
total	174 490	89 746 825	89,75	1 253	1 436

Contigs with B assignment confidences class 1 – 4 were used. Contigs with 'NA' did not assign to any of the wheat B subgenome reference sequences. chrUn: refers to sequences that are assigned to the B genome of wheat but lack a precise location on the chromosomes; clDNA: chloroplast DNA, mtDNA: mitochondrial DNA.

Supplementary Table 5. RepeatMasker overview dataset of total WGS assembly.

```
==================================================
file name: Aegilops_speltoides_WGSassembly.fasta
sequences:        1567708
total length:  1020055449 bp
GC level:         46.55 %
bases masked:   169546367 bp ( 17.56 %)
==================================================
               number of      length   percentage
               elements*    occupied  of sequence
--------------------------------------------------
Retroelements       523556    141902095 bp   13.91 %
   SINEs:              824        82139 bp    0.01 %
   Penelope              0            0 bp    0.00 %
   LINEs:            18305      4738538 bp    0.46 %
    CRE/SLACS            0            0 bp    0.00 %
     L2/CR1/Rex          0            0 bp    0.00 %
     R1/LOA/Jockey       0            0 bp    0.00 %
     R2/R4/NeSL          0            0 bp    0.00 %
     RTE/Bov-B           0            0 bp    0.00 %
     L1/CIN4         18305      4738538 bp    0.46 %
   LTR elements:    504427    137081418 bp   13.44 %
     BEL/Pao             0            0 bp    0.00 %
     Ty1/Copia      159659     42754041 bp    4.19 %
     Gypsy/DIRS1    344768     94327377 bp    9.25 %
       Retroviral        0            0 bp    0.00 %

DNA transposons      83652     13573243 bp    1.33 %
   hobo-Activator      748       107458 bp    0.01 %
   Tc1-IS630-Pogo    17995      1890616 bp    0.19 %
   En-Spm            46213      8331833 bp    0.81 %
   MuDR-IS905            0            0 bp    0.00 %
   PiggyBac              0            0 bp    0.00 %
   Tourist/Harbinger 11319      2349738 bp    0.23 %
   Other              7377       893598 bp    0.09 %

Rolling-circles          0            0 bp    0.00 %

Total interspersed repeats:   155475338 bp   15.24 %


Small RNA:             867        95392 bp    0.94 %

Satellites:          12796      3687684 bp    0.37 %
Simple repeats:     184455      8187164 bp    0.80 %
Low complexity:      38359      2100789 bp    0.21 %
==================================================

* most repeats fragmented by insertions or deletions
  have been counted as one element


The query species was assumed to be triticum aestivum
RepeatMasker version open-4.0.5 , default mode

run with cross_match version 1.090518
RepBase Update 20150807, RM database version 20150807
```

Supplementary Table 6. RepeatMasker overview dataset of B chromosome assigned contigs.

```
==================================================
file name: Aegilops_speltoides_B_chromosome.fasta
sequences:         174490
total length:    89572335 bp  (88714892 bp excl N/X-runs)
GC level:          45.99 %
bases masked:     6290926 bp ( 7.02 %)
==================================================
               number of      length   percentage
               elements*    occupied  of sequence
--------------------------------------------------
Retroelements        14265      4507876 bp    5.03 %
   SINEs:               53         5496 bp    0.01 %
   Penelope              0            0 bp    0.00 %
   LINEs:             1527       414722 bp    0.46 %
    CRE/SLACS            0            0 bp    0.00 %
     L2/CR1/Rex          0            0 bp    0.00 %
     R1/LOA/Jockey       0            0 bp    0.00 %
     R2/R4/NeSL          0            0 bp    0.00 %
     RTE/Bov-B           0            0 bp    0.00 %
     L1/CIN4          1527       414722 bp    0.46 %
   LTR elements:     12685      4087658 bp    4.56 %
     BEL/Pao             0            0 bp    0.00 %
     Ty1/Copia        6321      2276037 bp    2.54 %
     Gypsy/DIRS1      6364      1811621 bp    2.02 %
       Retroviral        0            0 bp    0.00 %

DNA transposons       4501       738915 bp    0.82 %
   hobo-Activator       33         4429 bp    0.00 %
   Tc1-IS630-Pogo      866        87005 bp    0.10 %
   En-Spm                0            0 bp    0.00 %
   MuDR-IS905            0            0 bp    0.00 %
   PiggyBac              0            0 bp    0.00 %
   Tourist/Harbinger   659       128021 bp    0.14 %
   Other (Mirage,        0            0 bp    0.00 %
    P-element, Transib)

Rolling-circles          0            0 bp    0.00 %

Unclassified:            2          204 bp    0.00 %

Total interspersed repeats:     5246995 bp    5.86 %


Small RNA:              67         7187 bp    0.01 %

Satellites:            533       157968 bp    0.18 %
Simple repeats:      15478       705256 bp    0.79 %
Low complexity:       3305       180300 bp    0.20 %
==================================================

* most repeats fragmented by insertions or deletions
  have been counted as one element


The query species was assumed to be triticum aestivum
RepeatMasker version open-4.0.5 , default mode
```

Complete (0B+B) and B assigned contigs. The RepeatMasker curated repeat library of wheat was applied to identify and annotate repetitive elements in *Ae. speltoides* based on sequence homology to its wheat relative.

Supplementary Table 7. Sequence similarity in B chromosome-derived sequences.

Dataset	SNP density	SNP count **	Total length [bp]	Count of contigs with SNPs	Count of contigs with overlap to homologous triplet
0B (0B seq.)	38	130,831	4.936.817	30,476	53,581
B chromosome	31	159,681	4.936.817	28,568	53,581
B genes*	42	339	14 294	75	95

* 229 B assigned genes

** including small INDELs and Q10 threshold for quality score at SNP site

Similarity was determined by calculation of SNP density in homologous triplets. These were identified by alignment of microdissected reads against the 0B genome assembly. Afterwards, B assigned genes were linked to homologous triplets to reveal SNP density in genic regions. Variants are detected in 0B genome assembly by sequence alignment of microdissected DNA from B chromosomes and WGS sequences from 0B.

Supplementary Table 8. Conditions used for TEM.

Freeze substitution	Temp [°C]	Time [h]
100% Aceton<br>+ 2% glutaraldehyde	-80	72
	-80 to -70	6
	-70	24
	-70 to -50	6
	-50	24
	-50 to -35	6
100% Aceton<br>+ 1% osmiumtetroxide	-35	24
	-35 to -10	6
100% Aceton	-10 to 20	6
Propylenoxide	20	0,2
**Resin embedding**	**Temp [°C]**	**Time [h]**
25% Spurr in propylenoxid	20	12
50% Spurr in propylenoxid	20	4
75% Spurr in propylenoxid	20	4
100 Spurr	20	12
Polymerization	70	24

Freeze substitution and resin embedding for ultrastructure analysis.

Supplementary Table 9. Details on quality improved sequence data.

dataset	sequencing method	type	reads [R1]	bp [R1]	reads [R2]	bp [R2]	Gbp	% total bp after quality processing	% total reads after quality processing
total microdiss. data	microdiss. Bs	MiDiSeq	6 414 893	440 827 766	6 414 893	442 012 289	0,88	53,33	36,70
B chromosome coverage *	microdiss. Bs	0B; +B					**1,55**		
WGS	WGS	0B	193 443 402	19 318 003 281	193 443 402	19 118 302 189	38,44	92,64	91,47
WGS	WGS	+B	210 453 030	21 009 568 832	210 453 030	20 772 589 378	41,78	91,78	90,56
total WGS data	WGS	0B; +B					**80,22**		
genome coverage**	WGS	0B; +B					**13,44**		

The table lists sequencing characteristics of pre-processed reads for microdissected and whole genome shotgun sequencing (0B and +B). '*' The chromosome coverage calculation is based on a chromosome size of 570 Mb using contamination removed, adapter cleaned and quality trimmed seq. data (R1 + R2). '**' The genome coverage calculation is based on a total genome size of 5.97 Gb (including B chromosome of 570 Mb) using adapter cleaned and quality trimmed WGS data (R1 + R2).

Supplementary Table 10. Primer sequences.

Primer name	Primer sequence
AesTR-183_F	GTCAAATGCGTGGGTCAAC
AesTR-183_R	GTTTGACCAAAGGACTTTCTCG
contig_416592_F	CATGCATGTGGAAGTGCGAG
contig_416592_R	CAGGGTGAAAGCTTGCACAC
contig_51504-1_F	CAAAACTGGAGCAGGGCATG
contig_51504-1_R	TTAAGGTTCCCGCACTCAGC
contig_148587_F	AGATGCCACTGGGAGTTGATG
contig_148587_R	GAGACTGGTGTGGGCTTGTC
contig_1343895_F	TGACTAGAGGTGGTTGAGTGC
contig_1343895_R	AGAGATGCTCTAAGGCAAAGCC
contig_1351371_F	CACAAACAGGGCAACAAGGG
contig_1351371_R	CAACATAAGCGGTCTCGACTG
contig_665257_F	GCTTGACCAAATCACCGTGG
contig_665257_R	TCGGCATGGCATGTTTTCAC
contig_235444_F	TGGTGGGACAAGAACATTTCG
contig_235444_R	CTGGAGCACCTGGTCTTCTG
contig_1043378-1_F	TGGTACCTAAATCACGCGGG
contig_1043378-1_R	CAGGCCGACCACAGTATGAC
contig_1043378-2_F	GTGCTCAGATAAATGACCGTCG
contig_1043378-2_R	AGGTAGTTCCTCGGCCTGG
contig_1021683_F	CCATCTCAAGGCAATCAATCATCAA
contig_1021683_R	TGGATTGTTTCTTGTTGTTTGATGA
ScCL11-1_F	AATGTTCCTTAGCTTGCCAGA
ScCL11-1_R	TTTTCGTGCTTCCATCAACC
AEGSP_Katzir_IPKv1_gene_25261-F	CTCTCCACCACGATCCC
AEGSP_Katzir_IPKv1_gene_25261-R	ACGAGTCGTAGCACAGCTC
AEGSP_Katzir_IPKv1_gene_29173-F	TGAAGTCCCTTAGCAATGAAG
AEGSP_Katzir_IPKv1_gene_29173-R	GCACATGTTCCCACTCCTC
AEGSP_Katzir_IPKv1_gene_5149-F	AGACCAGCTATTAACGTTGGC
AEGSP_Katzir_IPKv1_gene_5149-R	GTCGCAGCATCAAGGTC
AEGSP_Katzir_IPKv1_gene_5298-F	TCCATCTGTCAGGCATTC
AEGSP_Katzir_IPKv1_gene_5298-R	CATGCTTCTCCAATCCC
GAPDH-F	GAGTCTGCCCACCCATTCGTAA
GAPDH-R	GACATGCCATAGGTTTCAGCGAC
