# Supplementary material for: Supernumerary B chromosomes of Aegilops speltoides undergo precise elimination in roots early in embryo development
Source: Nat Commun. 2020 Jun 2;11:2764. doi: 10.1038/s41467-020-16594-x (PMC7265534; doi:10.1038/s41467-020-16594-x)
Supplement: Supplementary file 3 — Reporting Summary [file 41467_2020_16594_MOESM3_ESM.pdf]

nature research

Corresponding author(s): NCOMMS-19-34447, Houben

Last updated by author(s): May 6, 2020

Reporting Summary

Nature Research wishes to improve the reproducibility of the work that we publish. This form provides structure for consistency and transparency in reporting. For further information on Nature Research policies, see Authors & Referees and the Editorial Policy Checklist.

Statistics

For all statistical analyses, confirm that the following items are present in the figure legend, table legend, main text, or Methods section.

n/a	Confirmed	
☐	☒	The exact sample size (*n*) for each experimental group/condition, given as a discrete number and unit of measurement
☐	☒	A statement on whether measurements were taken from distinct samples or whether the same sample was measured repeatedly
☒	☐	The statistical test(s) used AND whether they are one- or two-sided<br>*Only common tests should be described solely by name; describe more complex techniques in the Methods section.*
☒	☐	A description of all covariates tested
☒	☐	A description of any assumptions or corrections, such as tests of normality and adjustment for multiple comparisons
☐	☒	A full description of the statistical parameters including central tendency (e.g. means) or other basic estimates (e.g. regression coefficient) AND variation (e.g. standard deviation) or associated estimates of uncertainty (e.g. confidence intervals)
☒	☐	For null hypothesis testing, the test statistic (e.g. *F*, *t*, *r*) with confidence intervals, effect sizes, degrees of freedom and *P* value noted<br>*Give P values as exact values whenever suitable.*
☒	☐	For Bayesian analysis, information on the choice of priors and Markov chain Monte Carlo settings
☒	☐	For hierarchical and complex designs, identification of the appropriate level for tests and full reporting of outcomes
☒	☐	Estimates of effect sizes (e.g. Cohen's *d*, Pearson's *r*), indicating how they were calculated

Our web collection on statistics for biologists contains articles on many of the points above.

Software and code

Policy information about availability of computer code

Data collection	No software was used to collect data
Data analysis	AgriGO was applied in version 2.0 for assignment of GO terms; Bedtools was applied in version 2.22.1 for sequence assessment of genomic regions; BLAST+ was applied in version 2.6.0 for sequence similarity analysis; BOWTIE was applied in version 2.2.9 for sequence read alignment; BWA was applied in version 0.7.17 for sequence read alignment; Circos was applied in version 0.67 for visualization of genomic regions; CLC assembly cell was applied in version 5.0.5 for genome assembly; Cutadapt was applied in version 1.9.1 for removing sequencing adapters; e!DAL was applied in version 2.7.0 for data submission and creation of DOIs; FastQC was applied in version 0.11.7 for FASTQ quality checks; GeMoMa was applied in version 1.4 for gene prediction; InterPro was applied in release version 66.0 for gene function assignment; Kmasker plants was applied in version 0.0.35 for k-mer ratio analysis; RepeatMasker was applied in version 4.0.8 for repeat detection; SAMtools was applied in version 1.3 for analysis of read alignments; Trimmomatic was applied in version 0.36 for quality trimming; VCFtools was applied in version 0.1.14 for variant calling. For flow-cytometry we used: FloMax Operating and Analysis Software Version 2.82, BD FACSTM Sortware Version 1.2.0.142, BD CellQuestTM Pro Version 6.1. For microscopy we used: ZENblack (Carl Zeiss GmbH), Imaris 8.0 (Bitplane), Photoshop CS5 (Adobe Systems).

For manuscripts utilizing custom algorithms or software that are central to the research but not yet described in published literature, software must be made available to editors/reviewers. We strongly encourage code deposition in a community repository (e.g. GitHub). See the Nature Research guidelines for submitting code & software for further information.

Data

Policy information about availability of data

All manuscripts must include a data availability statement. This statement should provide the following information, where applicable:

- Accession codes, unique identifiers, or web links for publicly available datasets
- A list of figures that have associated raw data
- A description of any restrictions on data availability

Genome data generated and analysed during the current study are available in the European Nucleotide Archive (ENA) under project numbers PRJEB29864 and PRJEB29862. Central data sets are maintained as DOIs in the PGP repository. The Aegilops speltoides WGS assembly is available under Data Citation 1. WGS contigs assignments and classifications are published under Data Citation 2. Repeat copy numbers are published under Data Citation 3. The functional annotation of candidate genes and the GO term enrichment analysis are published under Data Citation 4. Details for these DOIs (Data Citations 1 – 4, http://dx.doi.org/10.5447/ipk/2020/8, http://dx.doi.org/10.5447/ipk/2020/9, http://dx.doi.org/10.5447/ipk/2020/10, http://dx.doi.org/10.5447/ipk/2020/11) are given in the publication.

Field-specific reporting

Please select the one below that is the best fit for your research. If you are not sure, read the appropriate sections before making your selection.

☒ Life sciences ☐ Behavioural & social sciences ☐ Ecological, evolutionary & environmental sciences

For a reference copy of the document with all sections, see nature.com/documents/nr-reporting-summary-flat.pdf

Life sciences study design

All studies must disclose on these points even when the disclosure is negative.

Sample size	No specific sample size calculation was undertaken before experiments. We chose samples sizes according to standard practices in the field of cytogenetics.
Data exclusions	no data were excluded
Replication	All the microscopy experiments shown in the paper are representative of several replicates. The number of centromeres analyszed to determine the amount/level of CENH3 is reported in the paper.
Randomization	Not relevant for the study as genotypes or tretments were not compared with each other.
Blinding	blinding was not required as no genotypes and tretments were compared with each other

Reporting for specific materials, systems and methods

We require information from authors about some types of materials, experimental systems and methods used in many studies. Here, indicate whether each material, system or method listed is relevant to your study. If you are not sure if a list item applies to your research, read the appropriate section before selecting a response.

Materials & experimental systems

n/a	Involved in the study	
☐	☒	Antibodies
☒	☐	Eukaryotic cell lines
☒	☐	Palaeontology
☒	☐	Animals and other organisms
☒	☐	Human research participants
☒	☐	Clinical data

Methods

n/a	Involved in the study	
☒	☐	ChIP-seq
☐	☒	Flow cytometry
☒	☐	MRI-based neuroimaging

Antibodies

Antibodies used	polyclonal anti-CENH3 desbribed in Sanei et al. https://www.pnas.org/content/108/33/E498.short; monoclonal alpha tubulin https://www.sigmaaldrich.com/catalog/product/sigma/t5168?lang=de&region=DE&gclid=EAIaIQobChMIjd3o8fbI5QIVmeF3Ch1HagRDEAAYBCAAEgIYfvD_BwE Anti-mouse Alexa 488 (Molecular Probes, USA, cat. no. A11001, dilution 1:200) and anti-rabbit rhodamine (Jackson ImmunoResearch, USA, cat. no. 111-295-144, dilution 1:600) were used as secondary antibodies.
Validation	anti-grass CENH3 validated by Western blotting and indirect immunostaining see: https://www.pnas.org/content/108/33/E498.short;

anti-alpha tubulin validaed by Western and indirect immunostaining by SIGMAALDRICH https://www.sigmaaldrich.com/catalog/product/sigma/t5168?lang=de&region=DE&gclid=EAIaIQobChMIjd3o8fbI5QIVmeF3Ch1HagRDEAAYBCAAEgIYfvD_BwE

Flow Cytometry

Plots

Confirm that:

☒ The axis labels state the marker and fluorochrome used (e.g. CD4-FITC).

☒ The axis scales are clearly visible. Include numbers along axes only for bottom left plot of group (a 'group' is an analysis of identical markers).

☒ All plots are contour plots with outliers or pseudocolor plots.

☒ A numerical value for number of cells or percentage (with statistics) is provided.

Methodology

Sample preparation	Flow cytometric analysis of Ae. spetoides embryos<br>To analyse the proportion of B-chromosome-containing cells in 17 - 20 days old developing embryos, nuclei were isolated by manual chopping with a sharp razorblade using the CyStain PI Absolute P reagent kit (Sysmex-Partec, Germany) and absorption distributions were measured on a Sysmec CyFlow Space flow cytometer (Sysmex-Partec, Germany). Between 1300 and 6000 nuclei per embryo were analyzed.<br>Flow cytometric analysis of Ae. spetoides embryos<br>To analyse the proportion of B-chromosome-containing cells in 17 - 20 days old developing embryos, nuclei were isolated by manual chopping with a sharp razorblade using the CyStain PI Absolute P reagent kit (Sysmex-Partec, Germany) and absorption distributions were measured on a Sysmec CyFlow Space flow cytometer (Sysmex-Partec, Germany). Between 1300 and 6000 nuclei per embryo were analyzed.
Instrument	Sysmec CyFlow Space flow cytometer (Sysmex-Partec, Germany)
Software	FloMax Operating and Analysis Software for Flow Cytometry Particle Analysing Systems, Version 2.82
Cell population abundance	Based on the applied settings the abundance of nuclei within the crude suspensions was around 30%.
Gating strategy	Nuclei were separated from cellular debris by plotting the log-scale relative DNA content against log-scale SSC signals.

☒ Tick this box to confirm that a figure exemplifying the gating strategy is provided in the Supplementary Information.
