# Supplementary material for: Supernumerary B chromosomes of Aegilops speltoides undergo precise elimination in roots early in embryo development
Source: Nat Commun. 2020 Jun 2;11:2764. doi: 10.1038/s41467-020-16594-x (PMC7265534; doi:10.1038/s41467-020-16594-x)
Supplement: Supplementary file 4 — Description of Additional Supplementary Files [file 41467_2020_16594_MOESM4_ESM.pdf]

Description of Additional Supplementary Files

File name: Supplementary Data 1
Description: List of validated B chromosome genes. The table includes gene models of B located genes with corresponding contig IDs and chromosome locations in wheat B subgenome.

File name: Supplementary Data 2
Description: BLASTN results based on alignment of *Ae. speltoides* B-chromosome related genes against root-specific barley genes. B chromosome related genes of *Ae. speltoides* (229) were aligned against all transcript sequences of high-confidence genes of barley (IPK Barley BLAST Server) to identify root-specific genes. The first excel sheet represents the information about BLASTN results. The best BLASTN match for each of *Ae. speltoides* genes is shown. Additionally, information about expression values (Fragments per kilobase of exon model per million mapped reads, FPKM) in corresponding root specific genes of barley is provided in Supplementary Data 3.

File name: Supplementary Data 3
Description: Transcript isoforms expressed in barley according to the Barley Genome Explorer (Barlex). Expression profile of matching barley genes in different stages and tissues. Values are in FPKM (Fragments per kilobase of exon model per million mapped reads). EMB, 4-day embryos; ROO1, roots from seedlings (10 cm shoot stage); LEA, shoots from seedlings (10 cm shoot stage); INF1, young developing inflorescences (5mm); INF2, developing inflorescences (1-1.5 cm); NOD, developing tillers, 3rd internode (42 DAP); CAR5, developing grain (5 DAP); CAR15, developing grain (15 DAP); ETI, etiolated seedling, dark cond. (10 DAP); LEM, inflorescences, lemma (42 DAP); LOD, inflorescences, lodicule (42 DAP); PAL, dissected inflorescences, palea (42 DAP); EPI, epidermal strips (28 DAP); RAC, inflorescences, rachis (35 DAP); ROO2, roots (28 DAP); SEN, senescing leaves (56 DAP).

File name: Supplementary Movie 1
Description: Nondisjunction of centromere-active B chromosomes. Anaphase with lagging Bs after immunostaining of CENH3 (in purple) and α-tubulin (in green). Compare with Fig. 2.
